# A Buffered Local Anesthetic Without Epinephrine: Development, Characterization, and In Vivo Efficacy and Toxicity Analysis

**DOI:** 10.3390/pharmaceutics16081058

**Published:** 2024-08-12

**Authors:** Daniel Uzbelger Feldman, Billy B. Laun, Chirag Patel, Sonal V. Pande, Sai H. S. Boddu

**Affiliations:** 1 Department of Endodontology, Temple University Kornberg School of Dentistry, 3223 N. Broad Street, Philadelphia, PA 19140, USA; 2 Oral & Maxillofacial Surgery Carbondale, 1111 E. Walnut St Suite B, Carbondale, IL 62901, USA; 3 Department of Pharmacology, L. M. College of Pharmacy, Ahmedabad 380009, India; 4 Center of Medical and Bio-allied Health Sciences Research, Ajman University, Ajman P.O. Box 346, United Arab Emirates; s.boddu@ajman.ac.ae; 5 Department of Pharmaceutical Sciences, College of Pharmacy and Health Sciences, Ajman University, Ajman P.O. Box 346, United Arab Emirates

**Keywords:** dental anxiety, local anesthetic, lidocaine, epinephrine, buffered anesthesia

## Abstract

Lidocaine hydrochloride (HCl) 2% with 1:100,000 epinephrine (LW/E) is widely used to prevent pain during dental procedures and has been associated with injection sting, jittering effects, slow onset, and a bitter aftertaste. Since LW/E’s introduction in 1948, no significant modifications have been proposed. This study aims to design and characterize an improved dental lidocaine HCl injectable formulation without epinephrine (LW/O/E) via buffers, sweeteners, and amino acids. LW/O/E injections were prepared with pH and osmolality values of 6.5–7.0 and 590–610 mOsm/kg. Using the electronic tongue (ETongue), the LW/O/E injectable formulations were characterized for viscosity, injectability, and taste analysis. The results were compared with the LW/E control. In vivo efficacy and anesthetic duration of the samples were measured through radiant heat tail-flick latency (RHTFL) and hot plate (HP) tests and local toxicity was assessed after a single intra-oral injection in Sprague Dawley rats (SDR). The viscosity and injectability values of the LW/O/E samples were found to be comparable to the LW/E injection. ETongue taste analysis showed an improvement in bitterness reduction of the LW/O/E samples compared to the LW/E formulation. Toxicity studies of samples in SDR showed minor and transient signs of erythema/eschar and edema. Anesthetic duration via RHTFL and HP paw withdrawal latency time in SDR were found to be comparable for the LW/O/E Sample 3A and the LW/E injection (*p* < 0.05). In conclusion, the buffered, higher osmolality and reduced bitterness developed LW/O/E formulation (Sample 3A) could be considered a promising alternative to the LW/E formulation for dental use.

## 1. Introduction

Dental anxiety is common in the United States and 75% of patients report some level of dental phobia [1,2]. Dental anxiety can arise due to several factors, including past trauma during dental procedures, fear of the local anesthetic (LA) injection sting, fear of LA not working during the procedure, and the bitter taste of Las [3]. In this regard, anesthetic injection sting is attributed to 88.2% of dental fears yet, every year, 1.96 billion cartridges of dental LA are administered worldwide [4,5]

LAs form the backbone of pain control in dentistry and are the safest, most effective drug used in the prevention and management of dental pain. Lidocaine HCL, an amino amide-type compound, is one of the most widely used LAs. Chemically, lidocaine is 2-(diethylamino)-N-(2,6-dimethylphenyl) acetamide. Lidocaine was first synthesized in 1943, and it was introduced clinically in 1947. The addition of epinephrine in 1948 brought improvement in lidocaine’s duration, [6] though no significant improvements to the original formulation have been proposed until now.

The onset of dental pulpal anesthesia commonly occurs within 5 to 10 min and persists for approximately 60 min for 2% lidocaine HCl with 1:100,000 epinephrine (LW/E) as a vasoconstrictor [5]. Vasoconstrictors are widely used in LAs to increase the anesthetic duration [7]. Braun first demonstrated in 1903 that the addition of adrenaline to local anesthetics on injection prolonged the resultant anesthesia [8]. A major disadvantage of vasoconstrictors is the unwanted ‘jittering’ effect comprising temporary cardiac palpitations and tremors. In a clinical survey, 26% of the participants reported having at least one adverse reaction within the first two hours following the injection of LAs, with pallor, palpitations, diaphoresis, and dizziness being the most common adverse reactions reported [9]. Moreover, LAs containing vasoconstrictors sting on injection, due to the acidic pH (~3.3) required to stabilize epinephrine during storage.

The body buffers the acidic pH of the anesthetic solution closer to the physiologic range (7.35–7.45) before the anesthetic can begin to take effect [10]. The time that this transformation requires is a key factor in anesthetic latency. Increasing the pH of a cartridge of LW/E via sodium bicarbonate just before administering the injection results in several clinical advantages, including greater patient comfort during injection, more rapid onset of anesthesia, and decreased post-injection tissue injury [5,11]. In a recent double-blind, randomized, placebo-controlled, clinical crossover trial, a 3:1 ratio buffered 1% LW/E with sodium bicarbonate (674.6 mosmol/kg) was significantly less painful than the 9:1 ratio sample (467.1 mosmol/kg). Moreover, no serious adverse events occurred to the study participants [12]. Unfortunately, the drawback of decreasing injection sting via buffering is a requirement for additional armamentarium due to the chemical incompatibility and storage instability of sodium bicarbonate with lidocaine and epinephrine [13]. This additional gadget, an onset mixing pen and an onset cartridge connector to the LA cartridge, requires an extra pre-injection preparation step and increases supply costs.

In addition to injection sting, jittering effects, and slow onset of action, LA injections in dentistry struggle with bad aftertaste due to the lingering bitterness of the cartridge solution composition [3,14,15]. The attitude of patients has dramatically shifted to one in which they expect liquid medications to be pleasant and tolerable. This change in the patient’s perspective toward dental procedures can render oral care visits more pleasant [16].

Ringer’s saline solution was invented in the early 1880s by Sydney Ringer. In the 1930s, the original solution was further modified by American pediatrician Alexis Hartmann for treating acidosis. Hartmann added lactate, which mitigates changes in pH by acting as a buffer for acid [17]. Our proposed formulation will incorporate a lactated Ringer’s vehicle which buffers the pH within the cartridge to the physiologic range and metabolizes to sodium bicarbonate within the body [18], thus resulting in the elimination of the acidic sting, less tissue injury, and reduced latency. This proprietary improved formulation may eliminate the pre-injection buffering preparation steps and reduce cost [19].

Increasing plain LA formulation’s viscosity has shown similar duration effects when compared to their vasoconstrictor counterparts [20,21]. In addition, increasing serum osmolality is directly proportional to a vasoconstrictor effect [22,23]. As a result, our proposed formulation design will incorporate dextrose, a widely used ingredient in intravenous (IV) solutions for an increased viscosity, along with lactated Ringer’s and injectable amino acids for an incremented osmolality. This would increase the duration of anesthetic action without the need for epinephrine, thus reducing the unwanted jittering effects from an adrenaline rush.

The tertiary amine group of lidocaine HCl is considered responsible for its bitter and bad aftertaste. This problem may occur during pre- and post-injection dripping or after part of the injected volume refluxes back onto the oral cavity and the tongue [14,15,24]. Our innovative formulation will include bitterness suppressants comprised of the sweeteners dextrose and sodium saccharine, and bitter blockers L-arginine, glycine, and glutamic acid for an improved taste [19]. Dextrose/Lidocaine and Dextrose/Lactated Ringers are chemically stable and have been used together for years in medicine, the first for spinal anesthesia and the latter for intravenous fluid administration [19,25,26]. Dextrose Prolotherapy is presumed to work by several mechanisms, including a direct, osmotic, and inflammatory growth effect. Dextrose injections below a 10% solution directly stimulate the proliferation of cells and tissue without causing a histological inflammatory reaction. Dextrose is an ideal proliferant because it is water soluble and a normal component of blood chemistry, which can be injected safely into multiple areas and in large quantities [27]. Sodium saccharin is approved by the FDA for use in injectable formulations up to a concentration of 0.09% *w*/*v* [19]. By combining sweet-tasting amino acids or their salts with bitter drugs, it is possible to substantially reduce their bitterness [28]. Additionally, L-arginine is used to mask the bitter taste of quinine [29]. In vitro studies using an electronic tongue (ETongue) demonstrated that glutamic acid might be as effective as sucralose for diphenhydramine bitter taste blocking [30]. The taste of ampicillin improved markedly by preparing its granules with glycine, mixing them with sweeteners, and flavors, and finally compressing them into tablets [31].

Since 1948, when LW/E was introduced, few changes have transpired to improve the original formulation in dentistry. The study aims to design and characterize a buffered, increased osmolality, reduced bitterness lidocaine HCl injectable formulation without epinephrine (LW/O/E) for dental use. Initially, six different formulations were prepared and characterized for pH, osmolality, and stability at 40 °C. Based on the accelerated stability data, three variants of sample 3 were prepared and thoroughly characterized for viscosity, injectability, and taste analysis using the ETongue. The results were compared with the commercial 2% LW/E control. In vivo efficacy and anesthetic duration of the samples were measured through radiant heat tail-flick latency (RHTFL) and hot plate (HP) tests and local toxicity was assessed after a single intra-oral injection in Sprague Dawley rats (SDR).

## 2. Materials and Methods

### 2.1. Materials

Lidocaine hydrochloride monohydrate (LOT: SLCF3130) was procured from Sigma (St. Louis, MO, USA). Citric acid (LOT: V140772401), L-arginine hydrochloride (LOT: V14120300), hydroxypropyl-beta-cyclodextrin (LOT: V20022501), and L-glutamic acid (LOT: V20012803) were purchased from Bioworld (Dublin, OH, USA). Dextrose (Batch: 15510501) was purchased from Scharlau (Barcelona, Spain). Glutamic acid (Batch: 19827/1c) was purchased from Superchem Products Ltd. (Ipswich, UK) and saccharin sodium (Batch: G094906) was purchased from Loba Chewie Pvt. Ltd. (Mumbai, India). Hematoxylin, eosin, and formalin were obtained from Himedia (Maharashtra, India). Alpha-chloralose was purchased from Sisco Research Laboratories Pvt. Ltd., (Maharashtra, India), and urethane from Sigma Aldrich (Mumbai, India). Lactated Ringer solution was purchased from local pharmacies (City Life Pharmacy, University St-Al Jerf 1—Ajman, UAE).

### 2.2. Preparation of Various Combinations of LW/O/E Injections

Based on the preliminary observations, six different formulations were prepared. The selection of excipients and the concentration is based on the preliminary data and relevant literature evidence [24,32,33,34]. A proprietary procedure was followed for preparing a batch volume of 500 mL [35]. The osmolality of the solution was measured and adjusted (using dextrose) to 600 mOsm/kg using a Knauer K-7400S Semi-micro Osmometer (Berlin, Germany). The final solution was filtered using a 0.2 µm filter. The final preparation was filled in 10 mL glass vials and sealed with a rubber stopper and plastic aluminum flip caps (Chudeng, Hunan, China).

### 2.3. Analytical Method Development Using High-Performance Liquid Chromatography (HPLC)

A Shimadzu Prominence-i-LC-2030C 3D Auto Sampler (SIL-20A) HPLC system (Shimadzu, Kyoto, Japan) was used for the analysis of LW/O/E in formulations. The HPLC system was equipped with Shimadzu LC-20AT reciprocating pumps connected to a DGU 20A5 degasser with a CBM 20A integrator and SPD-M20A diode array detector. A Phenomenex Luna C18 column with a dimension of 150 × 4.6 mm, particle size of 5 µm, and pore size of 10 Å was used. The column was placed at 40 °C and the mobile phase comprising 20 mM phosphate buffer (pH 5.5) acetonitrile (74:26) was pumped at a flow rate of 1.5 mL/min. The injection volume was 10 µL. The absorbance of lidocaine HCl was measured at 230 nm, and the drug content in the samples was determined by plotting a calibration curve [36]. A stock solution of 1000 µg/mL of lidocaine HCl was prepared using methanol Merck (Darmstadt, Germany). The calibration standards, ranging from 10–50 µg/mL, were prepared in the mobile phase. Each calibration standard was analyzed in triplicate, and the average peak area was plotted against the amount of lidocaine HCl to obtain the calibration curve. The limit of detection (LOD) and limit of quantification (LOQ) were determined. The validation of the method was performed as per the ICH guidelines. Stress studies were performed to test the stability-indicating efficiency, i.e., the ability to effectively resolve the drug from its degradants, of the HPLC method. In 100 mL of various solutions (0.1 N HCl, 0.1 N NaOH, and 0.02% H_2_O_2_), 25 mg of lidocaine HCl was added. Approximately 10 mL of each solution in was placed in glass vials and stored at 60 °C. Stressed samples were analyzed using the HPLC method. After a predetermined time interval, lidocaine HCl peaks of stress samples were examined for any interference from degradant peaks.

### 2.4. Stability of Injectable Formulations

Stability studies were carried out for injectable formulations (Samples 1–6). Samples were placed in glass vials and stored at 40 °C for up to 6 months without light. Samples were taken at regular time intervals and measured for clarity, pH, osmolality, and drug content using HPLC. The clarity of formulation was measured by viewing the sample against a white and black background. The pH of formulations was measured using a universal multi-parameter portable meter ProfiLine pH/Cond 3320 (WTW, Weilheim, Germany). The pH meter was calibrated using standard buffer solutions of pH 4, 7, and 10 before pH measurements. About 150 µL of the sample was used for measuring the osmolality using a Knauer K-7400S Semi-micro Osmometer (Berlin, Germany) as per the manufacturer’s recommendation. The standards provided by the manufacturer were used as controls in the osmolality study. Samples were also stored at 25 °C without light and checked for clarity for up to 12 months.

### 2.5. Preparation of Optimized Formulations

Based on the stability data, sample 3 was found to contain an optimum combination of excipients for producing a stable formulation. Three variants of sample 3 were prepared as shown in Table 1. Samples 3A, 3B and 3C contained 2%, 1.9% and 1.5% LW/O/E, respectively. The drop in the osmolality due to the reduction in the drug content was compensated by dextrose or sodium saccharine as shown in Table 1. Lower concentrations of LW/O/E (1.9% and 1.5%) were prepared with the intention of assessing the possibility of achieving similar anesthetic activity at a lower dose compared to the commercial LW/E formulation.

### 2.6. Viscosity Determination

The viscosity of formulations was determined by using an Ostwald viscometer (PSL-Rheotek. Granger, IN, USA). The viscosity was determined by measuring the flow rate of samples 3A, 3B, and 3C through a capillary tube. At room temperature of 25 °C, each sample was added to the Ostwald viscometer with a pipette. A piece of rubber tubing was used to suck up the sample into the capillary arm of the viscometer until the surface of the liquid was above the upper mark. The liquid was then allowed to flow down the arm. The time required for the surface of the liquid to pass from the upper mark to the lower mark was noted. A commercial LW/E injection was used as a control. The viscosity of samples was determined using the equation below. All measurements were made in triplicate.

η_sample_ = (η_water_ × ρ_sample_ × t_sample_)/(ρ_water_ × t_water_)

where:

η_sample_ = Viscosity of sample
η_water_ = Viscosity of water
ρ_sample_ = Density of sample (measured using pycnometer)
ρ_water_ = Density of water (measured using pycnometer)
t_sample_ = Run time of sample from the upper mark to the lower mark
t_water_ = Run time of sample from the upper mark to the lower mark

### 2.7. Injectability Test

To evaluate the effect of hyperosmolality and viscosity on the injectability of samples, an injectability test was performed using a fully digital testing system with high precision control and accuracy with automated computer control of test method (Testometric M350-5 CT Tensile Tester, Testometric, Lancashire, UK). The tensile tester was used in a compression mode. Samples 3A, 3B, and 3C were used in the study and a commercial LW/E injection was used as a control. In brief, 5 mL of samples were loaded in a 10 mL plastic syringe (Becton Dickinson, Plymouth, UK) with a 27 G needle, or 0.5 mL was loaded in a 1 mL plastic syringe with a 30G needle (BD microfine, Franklin Lakes, NJ, USA). A clamp with grips attached to a stand was used to secure the chosen syringe. Alignment of the clamp grips with the center of the crosshead was ensured to achieve good data. Moreover, the clamps were secured firmly to prevent movement of clamps when a downward compression force was applied. A plastic cup was placed at the bottom plate to collect the extruded liquid. The injectability test was carried out at the crosshead speed of 1 mm/s, representative of clinical delivery to a patient. The average force required to sustain the movement of the plunger to expel the content of the syringe was measured (N), and it was recorded using comprehensive winTest™ Analysis universal Windows software (version 4.4.2). 

### 2.8. Taste Analysis Using ETongue

The taste was assessed using an Alpha MOS ASTREE ETongue system (Alpha MOS Inc., Hanover, MD, USA) equipped with an Alpha M.O.S. sensor set no. 2 (for pharmaceutical analysis) composed of seven specific sensors (ZZ, AB, GA, BB, CA, DA, and JE) on a 48-position autosampler using 25-mL beakers. All the data generated on the ASTREE system were treated using multidimensional statistics on AlphaSoft V14 software. Taste analysis was conducted for samples 3A, 3B, and 3C. Distilled water (S1) and commercial LW/E ‘Octocaine^®^-100’ (S2) (Novocol, Cambridge, ON) were used as controls. Twenty milliliters of each sample were placed directly into a beaker and analyzed by the Alpha MOS ASTREE ETongue. Acquisition time and time per analysis were set at 120 and 180 s, respectively. The ETongue signal of each solution was measured at equilibrium using seven sensors (ZZ, AB, BA, BB, CA, DA, and JE). Solutions were analyzed in triplicate. ASTREE sensors were cleaned with deionized water between measurements. The distance on the taste map between FC and other formulations was measured by the Alpha MOS ASTREE ETongue.

### 2.9. Local Toxicity and Efficacy Studies

#### 2.9.1. Animals

Sprague Dawley rats (SDR) of either sex, weighing 200–250 g, were used in the study. The animals were kept at the laboratory animal house of Lallubhai Motilal (L. M.) College of Pharmacy, Ahmedabad, India. The animals were maintained under standard environmental conditions and were allowed free access to feed and water ad libitum. All the procedures were conducted following the Committee for the Purpose of Control and Supervision of Experiments on Animals (CPCSEA) guidelines. The study protocol (LMCP/IAEC/2023/2/0095) was approved by the L. M. College of Pharmacy, Ahmedabad, India. Rats were housed in standard polypropylene cages within the laboratory under controlled conditions: a 12-h light/dark cycle, temperature maintained at 22 ± 3 °C, 30–70% RH, and continuous air exhaust. Food and water were provided ad libitum.

#### 2.9.2. Local Toxicity of LW/O/E Formulations in Sprague Dawley Rats

One or two days before test item administration, the animals were randomized based on their body weights and allocated into seven groups, G1, G2, G3, G4, G5, G6, and G7, with 15 animals in each group. G1: Saline 0.9%, which has osmolality similar to body fluids (negative control); G2: Marketed LW/E Injection (Octocaine-LW/E injection, USP, Novocol, Cambridge, ON, Canada); G3: Dextrose 5% and Sodium Chloride (406 mOsm/L, Otsuka DNS, Ahmedabad, India); G4: Dextrose 10% *w*/*v* Solution (505 mOsm/L, Otsuka DNS, Ahmedabad, India); G5: Sample 3A—2% LW/O/E (600 mOsm/kg); G6: Sample 3B—1.9% LW/O/E (600 mOsm/kg); G7: Sample 3C—1.5% LW/O/E (600 mOsm/kg).

The animals received the formulations in the oral mucosa (6.6 mg/kg dose) of the upper right first molar and the left side was considered as control. The dose of 6.6 mg/kg was calculated considering the therapeutic dose of lidocaine to be 3.5 mg/kg in humans. The dose for rats was calculated based on body weight using a conversion factor of 6.2. Considering the average weight of rats as 0.3 kg, 0.1 mL of 2% drug solution will result in a dose of 6.6 mg/kg. A 26G and 1.5” needle attached to a 1 mL syringe (Dispovan, New Delhi, India) was used for injecting formulations. At the commencement of the study, the weight variation of animals was minimal and did not exceed ±20% of the mean weight of each sex. All the animals were examined for signs of erythema and edema, and the responses were scored at 1 h, 6 h, 24 h, and 4 days. Reactions were graded and recorded as per Draize’s protocol [37]. Scores for erythema and eschar formation were as follows: no erythema (0), very slight erythema (1), well-defined erythema (2), moderate to severe erythema (3), severe erythema to eschar formation (4). Scores for edema formation were as follows: no edema (0), very slight edema (1), slight edema (2), moderate edema (3), severe edema (4). Animals were sacrificed under anesthesia (urethane 1 g/kg and alpha-chloralose 50 mg/kg) for 6 h (*n* = 5 animals), 24 h (*n* = 5 animals), and 4 days (*n* = 5 animals) under each group, and the maxillae along with soft tissues were removed, weighed, and preserved in 10% formalin. Maxillae, along with soft tissues, were processed for histopathological evaluation. The processed tissues were embedded in paraffin and thin sections of 3–5 μ thickness were made. The sections were placed on a clean grease-free slide and stained with hematoxylin and eosin. The cross-sections were qualitatively analyzed to evaluate the intensity of the leucocitaria infiltration and/or any area of necrosis. The cross-sections were photographed by a photomicroscope (Magnus, MX21i-B LED Binocular. New Delhi, India). The analyzed regions were the site of the injection and surrounding connective tissue in the most internal portion of the anterior maxillary fornix. A qualitative score of the local tissue inflammation was measured based on the following descriptions: no infiltrate (1), minimal infiltrate (2), mild infiltrate (3), severe infiltrate (4), and severe infiltrate with necrosis areas (5). Histopathological evaluation was performed by a pathologist in a blinded approach on stained sections obtained from animals.

#### 2.9.3. Efficacy Studies of LW/O/E Formulations in Sprague Dawley Rats

One or two days before test item administration, the animals were randomized based on their body weights and allocated into five groups, G1, G2, G3, G4, and G5, with 6 animals in each group. G1: Saline 0.9%, which has osmolality similar to body fluids (negative control); G2: Marketed LW/E Injection (Octacaine (Lidocaine and Epinephrine Injection, USP); G3: Test 1: Sample 3A—2% LW/O/E (600 mOsm/kg); G4: Sample 3B—1.9% LW/O/E (600 mOsm/kg); G5: Sample 3C—1.5% LW/O/E (600 mOsm/kg). All formulations were administered with a 26-gauge needle connected to a micro syringe (DispoVan, India), 0.05 mL of the drug was injected near the dorsal or lateral surface of the tail in the RHTFL test, whereas, in the HP test, the drug was injected on the planta (dose volume maximum up to 0.1 mL).

#### 2.9.4. Radiant Heat Tail-Flick Latency (RHTFL) Test

Animals without any pre-medication were placed in transparent polypropylene chambers (Orchid Scientific, Nashik, India), and then, the middle regions of the tails were exposed to a thermal light source (a single-fixed aperture) (Orchid Scientific, Nashik, India). The time-lapse from the onset of irradiation to the time when the animals moved their tails away from the thermal source was measured and named TFL, representing the sensitivity of the animals to temperature. The stimulatory radiation intensity was set to 75 to make the TFL of most SDR between 2 and 5 s. Each rat’s baseline TFL was determined one day before the experiment. Animals with a basic value of 5 s were not included. The cut-off time was set to 10 s, i.e., the radiation did not last more than 10 s to prevent tissue damage. If no tail-flick occurred after 10 s, TFL was recorded as 10 s. Tail-flick latencies in treated animals were measured at 0, 1, 2, 3, 4, 5, 10, 20, 30, 40, 50, 60, 90, 120, 150, 180, 210, 240, and 270 min after drug administration using a Tail-flick Analgesiometer (Orchid Scientific & Innovative. Nashik, India).

#### 2.9.5. Radiant Heat Hot Plate (HP) Test

The antinociception ability of formulations was also assessed in SDR using the HP test. After injection of formulations on the planta, SDR was placed on a hot plate (50 °C). Response time for observed behavioral changes, like paw licking, stomping, jumping, and escaping from the hot plate, was recorded to examine the normal heat pain threshold before treatment and the pain threshold after treatment. The cut-off time for the HP test was 15 s. Response times were measured at 0, 1, 5, 10, 20, 30, 40, 50, 60, 90, and 120 min after drug administration.

### 2.10. Statistical Analysis

Statistical analysis was performed using Graph-pad prism, version 7.04 software. All statistical tests were performed at a 95% confidence interval and a *p* < 0.05 was considered significant.

A two-way analysis of variance was used to compare the groups. The results of all observations under study were expressed as Mean ± Standard Deviation (SD) where applicable in the form of summary tables.

## 3. Results and Discussion

All samples 1–6 were found to be clear and colorless at varying concentrations of excipients. The pH of formulations was maintained between 6.5 and 7.0 with sodium hydroxide, and osmolality was adjusted to 600 mOsm/kg using dextrose. Lidocaine is a weak base and exhibits pH-dependent solubility, with good solubility at acidic pH. A 2% solution of lidocaine HCl precipitates around pH 5.7 due to insufficient solubility [38,39]. The commercial formulation of LW/E is maintained at a pH of 4.5 (3.3–5.5), which provides the required solubility of lidocaine and stability of epinephrine. However, an acidic solution is painful upon intradermal or subcutaneous injection and pain can be reduced by buffering the solution towards the physiological pH of 7.4. In the present study, lidocaine HCl solubility was achieved through the careful selection of excipients. The drug content in the samples was measured using a validated HPLC method. The retention time of the drug was found to be at 4.622 min. The assay method was found to be linear in the range of 10–50 µg/mL with a good correlation coefficient value. The percentage recovery of lidocaine HCl ranged from 99.62% to 100.62%. The intraday precision (measured by %RSD) was found to be in the range of 0.27% to 0.95%. The percentage recovery of lidocaine ranged from 99.87% to 103.20%. Stability-indicating assay was conducted to assess the suitability of developed HPLC by stress testing the drug under various conditions (acidic, basic, and oxidative conditions). Less than 5% degradation of the drug occurred in 0.1 N hydrochloric acid (HCl) and 0.1 N sodium hydroxide (NaOH) samples. In the case of stability-indicating samples in 0.02% hydrogen peroxide (H_2_O_2_) a significant degradation (~25%) was observed. However, no interference between the drug and degradant peaks was observed. Similar results were reported by Bhusal et al. [36].

**Figure 1:**
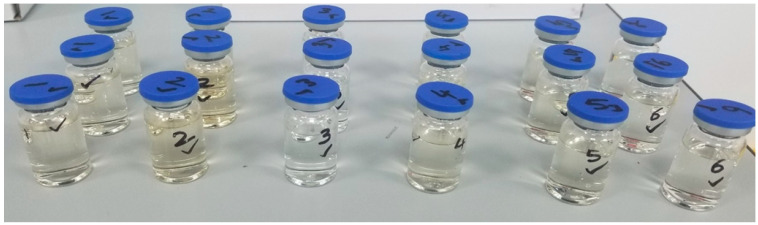
Samples stored at 40 °C without light showed discoloration after 4 months.

Samples were continuously monitored for any color changes. The initial pH of samples was found to be between 6.5 and 7.0 with osmolality ranging between 590 and 610 mOsm/kg. Samples were found to be stable for up to 4 months concerning clarity, pH, osmolality, and drug content. However, discoloration was observed in all samples after 4 months, except Sample 3 (Figure 1).

Despite discoloration, parameters such as pH, osmolality, and drug concentration were unchanged. The drug content values ranged between 90 and 110% through the stability period. The discoloration increased after 6 months. The discoloration could be due to the interaction between dextrose and glycine [40]. In Sample 3, the presence of citric acid prevents the interaction, and thus Sample 3 remained clear. Samples stored at 25 °C did not show any signs of discoloration for up to 12 months. This indicates that temperature acts as a catalyst in the interaction of dextrose and glycine. Stability data indicated that Sample 3 has the right combination of excipients for producing a stable formulation. Three variants of Sample 3 (3A, 3B, and 3C) were prepared as shown in Table 1 and further characterized for viscosity, injectability, in vitro release, taste analysis using ETongue, and toxicity and efficacy studies in rats. Sample 3A contained 2% LW/O/E, like the commercial LW/E formulation. However, Samples 3B and 3C contained only 1.9% and 1.5% LW/O/E, respectively. Lower concentrations of LW/O/E were prepared with the intention of assessing the possibility of achieving similar anesthetic activity at a lower dose compared to the commercial formulation and for developing a potential pediatric dentistry formulation.

The viscosity of the injectable formulation is an important parameter as it impacts the formulation injectability. Samples 3A, 3B, and 3C have a higher osmolality of 590–600 mOsm/kg when compared to the marketed formulation (~300 mOsm/kg). Despite higher osmolality, the viscosity values of Samples 3A, 3B, and 3C were found to be similar to the marketed LW/E injection and increased proportionally to the dextrose concentration. The viscosity values are presented in Table 2. The injectability of different samples was compared with the marketed LW/E formulation and distilled deionized water. Injectability is defined as the force/pressure required for injection or the evenness of flow through the syringe needle [41]. Generally, 27G and 30G needles are most commonly used in dentistry [42]. Hence, we considered these size gauge needles in our study. The results of the injectability study are presented in Table 2.

Water was used as a control in the study. The injectability values of commercial formulation, samples 3A, 3B, and 3C were found to be 19.45 ± 0.35 N, 19.20 ± 0.01 N, 19.85 ± 0.07 N, and 20.13 ± 0.23 N, respectively with a 10 mL syringe/27G needle. The injectability values of samples 3A, 3B, and 3C were found to be close to the injectability value of the commercial formulation. The recommended injectability value of parenteral should be less than 20 N [43]. All samples 3A, 3B, and 3C were in agreement with above the recommended value. As expected, a higher force is required to eject samples from a 10 mL syringe with a 27G needle when compared to a 1 mL syringe with a 30G needle. It is understood that the injectability of formulations depends on viscosity. Despite a marginal increase in the viscosity of samples 3A, 3B, and 3C compared to the marketed formulation, the injectability values are close, indicating no ejection difficulty from the syringe via a needle to the injection site [41]. In a viscosity injection pain study, viscosity levels had a significant impact on perceived injection pain (*p* = 0.0003). Specifically, less pain was associated more with high viscosity (VAS = 12.6 mm) than medium (VAS = 16.6 mm) or low (VAS = 22.1 mm) viscosities, with a significant difference between high and low viscosities (*p* = 0.0002). [44]

**Figure 2:**
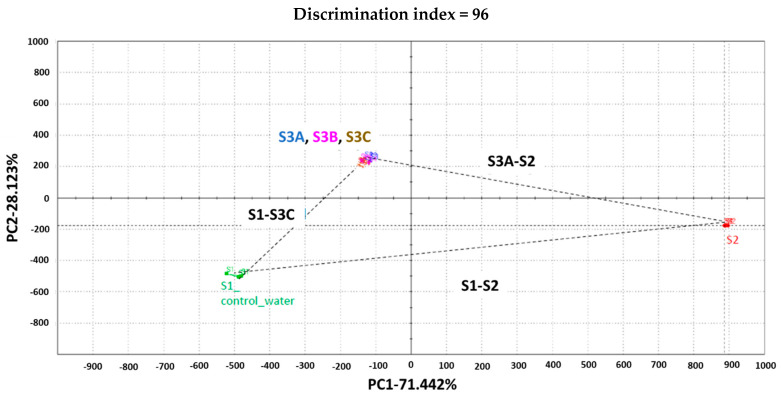
Taste map based on principal component analysis (PCA) with AHS, PKS, ANS, and SCS sensors. S1: water, S2: 2% lidocaine HCl with 1:100,000 epinephrine (LW/E), S3A: Sample 3A, S3B: Sample 3B, S3C: Sample 3C.

The taste analysis was conducted by a third party (Alpha MOS ASTREE ETongue, Hanover, MD, USA). Based on our experience, ETongue measurement for taste analysis was found to be precise and accurate. Human subjects can be used in taste analysis. However, it is time-consuming and expensive, and there is a possibility of intra- and inter-subject variability. Hence, we used ETongue for taste analysis in the current study. Taste analysis was conducted for samples 3A, 3B, and 3C. Distilled water (S1) and 2% LW/E ‘Octocaine^®^-100’ (S2) were used as controls. The signal of each sensor on each assay was integrated into a matrix of data that was computed by multidimensional statistic tools. A taste map based on Principal Component Analysis (PCA) was generated using all sensors. There is a clear difference between the formulations (Figure 2).

S1 and S2 (commercial LW/E) are well discriminated against along PC1 which represents 71%. Samples 3A, 3B, and 3C were found to be closer to S1 than to S2. The Euclidian distance between formulations was calculated to assess taste proximity between samples: the lower the distance, the closer the taste. A Discrimination Index (DI in %) was also determined for each pair of samples. This indicator considers the average difference between the pairs to compare, as well as the dispersion of each sample. The closer the index to 100%, the greater the distance between the centers of gravity and the smaller the dispersion within groups. The DI can help then to assess the significance of differences between the groups. S1 and S2 are the most distinct samples with distance [S1, S2] = 1424 and DI > 90%. These data suggest significant taste differences between formulations S1 and S2. S3A, S3B, and S3C are closer to each other from the five analyzed formulations, with distances [S3A, S3B] = 51; [S3A, S3C] = 116; [S3B, S3C] = 65. Distances [S2, S3A]; [S2, S3B] or [S2, S3C] are between 1096 and 1105 with DI > 90%. This information revealed significant taste differences between formulation S2 and formulations S3A, S3B, or S3C. Distances [S1 and S3A, S3B or S3C] < distances [S2 and S3A, S3B or S3C]. This suggests that S3A, S3B, and S3C are closer to S1 than to S2.

**Figure 3:**
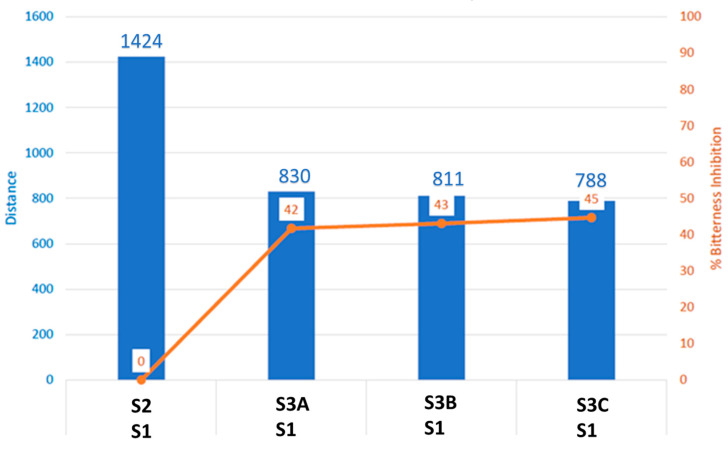
Percent bitterness prediction. S1: control water, S2: 2% lidocaine HCl with 1:100,000 epinephrine (LW/E), S3A: Sample 3A, S3B: Sample 3B, S3C: Sample 3C.

To compare the bitterness/sweetness of S3A, S3B, and S3C with the commercial formulation (S2), the group distance [S1, S2] = 100 with a 100% bitterness perception level was considered. The formula = [S1, S3A] * 100/[S1, S2] was applied to estimate % bitterness for groups [S1, S3A]; [S1, S3B] or [S1, S3C]. The percent bitterness inhibition = 100% − % bitterness perception. Figure 3 suggests that about 42, 43 and 45% bitterness are masked respectively in samples S3A, S3B, and S3C. The sweetness is inversely proportional to the bitterness (with S1 and S2 as controls), then the sweetness of formulations was ranked as S2 < S3A, S3B < S3C (Figure 3).

The repeatability of the measurements on Astree ETongue was determined for each sample on 3 replicates. The mean and standard deviation values are shown in Table 3. The results are comparable. The sourness/saltiness of all samples were compared. Direct sourness and saltiness ranking via the taste screening module of Alpha Soft predicted S2 as the sourest formulation and S1 as the least salty formulation.

Preferably, injectables should be isotonic (osmolality~300 mOsm/kg) with a pH close to the physiological pH to minimize pain, irritation, and tissue damage. Studies indicate that hyperosmolar solutions (up to 600 mOsm/kg) are well tolerated provided the right injection technique and the composition are maintained [45]. In our study, the optimized Samples 3A, 3B, and 3C were maintained at a pH of 6.5–7.0 with osmolality ranging between 590 and 610 mOsm/kg.

The toxicity of Samples 3A, 3B, and 3C was ascertained in rats. In this study, independent variables and sources of error, such as variations in local circulation, absorption, and metabolism in case of local inflammation, and fever were not considered. A single-dose injection in the oral mucosa of the upper right first molar in SDR was considered effective in assessing the toxicity of Samples 3A, 3B, and 3C. Local tolerability was evaluated based on the severity of signs of erythema and eschar, edema, and histological evaluation. Draize’s method was used to observe the signs of erythema and eschar following the injection of control and samples. Hyperosmolar solutions of dextrose 5% with sodium chloride (406 mOsm/L) (D5NS) and dextrose 10% (D10) *w*/*v* solution (505 mOsm/L) were also used as controls in this study, along with 0.9% saline and commercial LW/E. D5NS is administered intravenously as a parenteral replenishment fluid, while D10 *w*/*v* solution is administrated as intravenous infusions as a source of water and calories.

After 6 h, 24 h, and 96 h, erythema and eschar formation were evaluated and scored according to Draize’s scoring criteria. No signs of erythema and eschar formation were observed in the commercial formulation. However, Samples 3A, 3B, and 3C showed very slight erythema to well-defined erythema at 6 h that disappeared after 24 h (Table 4).

**Figure 4:**
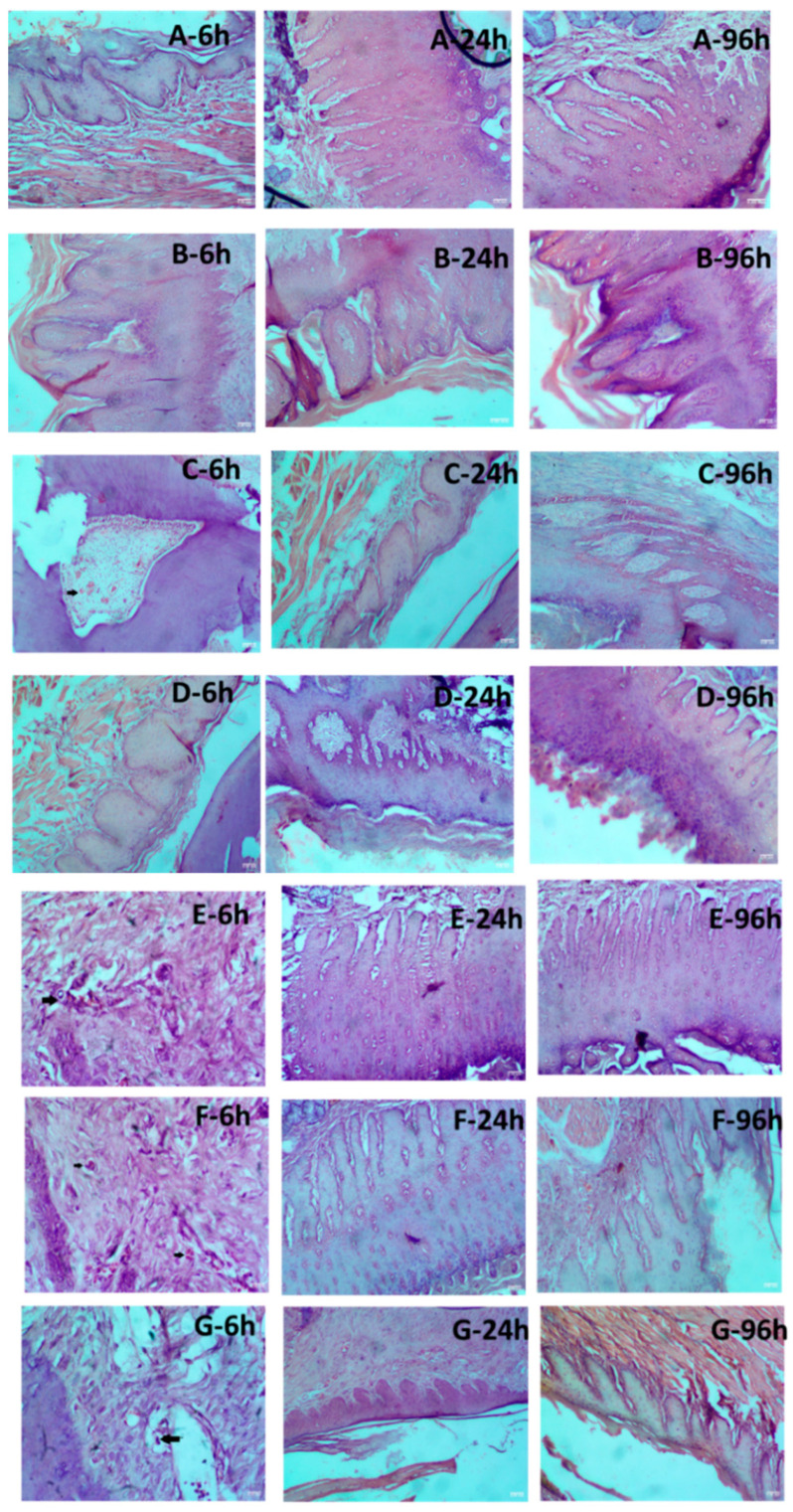
Histological evaluation of hematoxylin and eosin staining in the oral mucosa of rats injected with various formulations (**A**) 0.9% Saline solution; (**B**) Commercial 2% lidocaine HCl with 1:100,000 epinephrine; (**C**) Dextrose 10% *w*/*v* Solution (505 mOsm/L); (**D**) Dextrose 5% and Sodium Chloride 0.45% Injection (406 mOsm/L); (**E**) Sample 3A; (**F**) Sample 3B; (**G**) Sample 3C. Oral mucosa tissue sections of each mouse were stained with HE at 6, 24, and 96 h of injection in SD rats. Infiltration of leucocytes is indicated by black arrows (**C**)—6 h, (**E**)—6 h, (F)—6 h, and (**G**)—6 h. Scale bar in each image = 50.

From these results, it was apparent that solutions with higher osmolality tend to cause erythema and eschar, which are transient. Edema scores were used to observe the swelling after injection and dissipation of the bleb. No edema formation was observed in SDR following the injection of control and test formulation after 6 h, 24 h, and 96 h, except for Sample 3B, wherein slight edema was observed at 6 h and resolved at the later time points. D5NS and D10 *w*/*v* solution showed no significant signs of erythema/eschar and edema in comparison to 0.9% saline solution. The toxicity of test Samples 3A, 3B, and 3C were compared against D5NS and D10 *w*/*v* solution. Interestingly, no significant signs of erythema and edema were noticed between D10 *w*/*v* solution and Sample 3A, 3B, and 3C. However, concerning D5NS, some signs of very slight erythema to well-defined erythema were noted for Samples 3A and 3B at 6 h, which gradually disappeared. The histological sections of maxilla bones along with soft tissues were observed under a microscope by an experienced pathologist, and tissue changes were analyzed in sections of the samples exposed to the control and test formulations. Figure 4 shows the histological sections of groups G1, G2, G3, G4, G5, G6, and G7. In G1 and G2, injected with saline 0.9% water and commercial formulation, there were no changes. Slight changes ranging from minimal to mild infiltration were visible in rats injected with D10 *w*/*v* solution, Samples 3A, 3B, and 3C at 6 h and resolved at the later time points.

**Figure 5:**
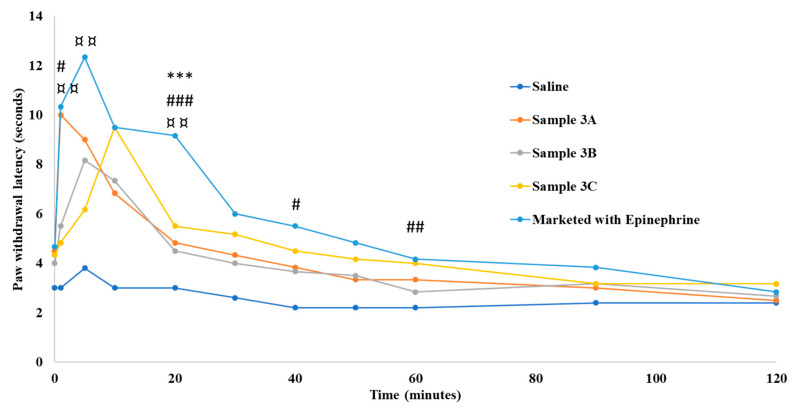
Paw withdrawal latency time in Sprague Dawley rats. “*”, “#”, and “¤” indicate statistically significant differences between the marketed 2% lidocaine HCl with 1:100,000 epinephrine and samples 3A, 3B, and 3C at the respective time points. Values are shown as mean of *n* = 6 samples. # *p* < 0.01 moderate difference; ## *p* < 0.01 strong difference; ¤¤ *p* < 0.01 strong difference; *** *p* < 0.001 very strong difference; ### *p* < 0.001 very strong difference.

The efficacy of test Samples 3A, 3B, and 3C was compared with the commercial LW/E using the HP sensory test and RHTFL test in an SDR model. In the HP sensory test, the commercial formulation showed a maximum reaction time of 12.33 s at 5 min, after which the effect gradually declined in about 60 min. A similar trend was observed in Sample 3A with a maximum reaction time of 10 s at 1 min and a gradual decline in about 40 min. No significant difference in the reaction time was observed between the commercial formulation Sample 3A, except for the time point of 20 min (Figure 5).

**Figure 6:**
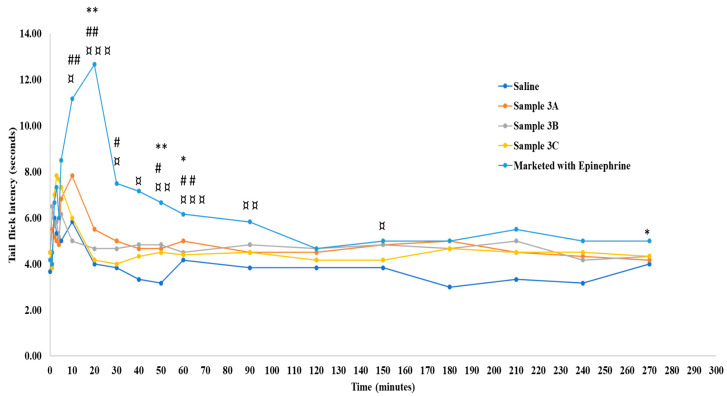
Radiant heat tail-flick latency time in Sprague Dawley rats. “*”, “#”, and “¤” indicate statistically significant differences between the marketed 2% lidocaine HCl with 1:100,000 epinephrine and samples 3A, 3B, and 3C at the respective time points. Values are shown as mean of *n* = 6 samples. # *p* < 0.01 moderate difference; * *p* < 0.01 moderate difference; ¤ *p* < 0.01 moderate difference; ** *p* < 0.01 strong difference; ¤¤ *p* < 0.01 strong difference; ## *p* < 0.01 strong difference; ¤¤¤ *p* < 0.001 very strong difference.

Despite the absence of epinephrine in Sample 3A, the anesthetic activity was comparable to the commercial formulation. This could be attributed to the vasoconstriction associated with the hyperosmolar (~600 mOsm/kg) Sample 3A [46,47,48]. A recent study published by Fleury et al. concluded that hyperosmolarity leads to solvent outflux, shrinkage, and subsequent vascular collapse [23]. It is well known that epinephrine in commercial formulations acts as a vasoconstricting agent and thus extends the duration of action [49]. Through this study, it is evident that the hyperosmolality of Sample 3A could replace the vasoconstrictor effect of epinephrine. However, the overall performance of Samples 3B and 3C were slightly lower than the commercial formulation at a majority of time points. Similar results were obtained using the RHTFL test. More frequent reaction time points were studied using this method and the control basal reaction time of SDR in RHTFL tests was found to be around 4.1 s. The commercial formulation showed a maximum reaction time of 12.6 s at 20 min, after which the effect was observed for up to 270 min. No significant difference in the reaction time was observed between the commercial formulation and Sample 3A, except for the time points of 20, 50, and 60 min (Figure 6).

However, a significant difference in reaction times was noted at 10, 20, 30, 40, 50, and 60 min for Samples 3B and 3C in comparison with the commercial formulation. In summary, the increase in the formulation’s viscosity and osmolality via dextrose, lactated Ringer’s, and amino acids incorporation did not have an impact on its injectability, but a there was a statistical significance in terms of anesthetic duration comparable to that of the marketed LW/E.

## 4. Conclusions

In conclusion, we have prepared and characterized a buffered, increased osmolality and reduced bitterness 2%, 1.9%, and 1.5% LW/O/E for dental use in terms of in vitro stability, viscosity, injectability, and taste analysis, as well as in vivo efficacy, anesthetic duration, and toxicity. The final pH and osmolality values were maintained between 6.5 and 7.0 and 590–610 mOsm/kg, respectively. Despite higher osmolality, the viscosity and injectability values of Samples 3A, 3B, and 3C were found to be similar to the marketed LW/E injection. Based on the data generated by the ETongue in terms of PCA Euclidian distance and Discrimination Index, Samples 3A, 3B, and 3C showed a 42, 43, and 45% reduction in the bitterness in comparison with the commercial LW/E formulation. Toxicity studies of optimized samples in SDR showed only minor signs of erythema/eschar and edema in the initial time point and disappeared after 6 h. The paw withdrawal latency time and RHTFL time in SDR were found to be comparable for Sample 3A containing 2% LW/O/E and commercial LW/E injection (*p* < 0.05). In conclusion, the buffered, higher osmolality and reduced bitterness formulation (Sample 3A) without epinephrine could be considered as a promising alternative to the commercial formulation with epinephrine for dental use and more research will be performed to evaluate this further. In dental practice, an LW/O/E formulation that can decrease injection sting, anesthetic onset, jittering effects, and bad aftertaste while maintaining duration could have a dramatic positive impact on public health and dental anxiety, while providing a distinctive advantage to the pharmaceutical manufacturer that offers it.

## Figures and Tables

**Table 1:** Variants of Sample 3 with varying drug and excipient concentrations.

Ingredients	Sample 3A	Sample 3B	Sample 3C
Lidocaine hydrochloride monohydrate (g)	5.332	5.065	3.999
Sodium saccharine (g)	0.225	0.225	0.263
Dextrose (g)	5	5.25	6
Glutamic acid (g)	1.25	1.25	1.25
Glycine (g)	0.5	0.5	0.5
Citric acid (g)	0.25	0.25	0.25
L-arginine	0.5	0.5	0.5
Water for injection (ml)	50	50	50
10 N Sodium hydroxide (to adjust pH 6.7–7.0)	1.3	1.4	1.3
Lactated Ringers (mL)	QS 250	QS 250	QS 250
Osmolality (mOsm/kg)	590–610	590–610	590–610

**Table 2:** Viscosity values of samples were measured using an Ostwald viscometer of *n* = 3 samples and injectability study results of optimized formulations and the marketed 2% lidocaine HCl with 1:100,000 epinephrine (LW/E). Values are represented by a mean ± standard deviation (SD) of *n* = 2 samples.

Sample	Viscosity (cps) (Mean Value ± SD) *n* = 3	10 mL Syringe with 27G Needle (Newtons)	1 mL Syringe with 30G Needle (Newtons)
Marketed LW/E	1.142 ± 0.006	19.45 ± 0.35	2.12 ± 0.16
Sample 3A	1.157 ± 0.006	19.20 ± 0.01	1.68 ± 0.01
Sample 3B	1.167 ± 0.012	19.85 ± 0.07	2.78 ± 0.16
Sample 3C	1.213 ± 0.008	20.13 ± 0.23	3.87 ± 0.53

**Table 3:** Standard deviation (SD) and relative standard deviation (RSD) for experimental and control samples including the marketed 2% lidocaine HCl with 1:100,000 epinephrine (LW/E).

Code	SD	%RSD
S1—Water	12.58	1.57
S2—Marketed LW/E	11.49	1.36
S3A—Sample 3A	5.06	0.58
S3B—Sample 3B	4.75	0.78
S3C—Sample 3C	4.43	0.53

**Table 4:** Toxicity study data of formulations in Sprague Dawley rats at various time points. “*” indicates statistically significant differences between the marketed 2% lidocaine HCl with 1:100,000 epinephrine (LW/E) formulation and samples 3A, 3B, and 3C. Values are shown as mean ± standard deviation (SD) of *n* = 5 samples.

	Erythema and Eschar Formation			Edema Formation			Histology		
Samples/Time Points	6 h	24 h	96 h	6 h	24 h	96 h	6 h	24 h	96 h
0.9% Saline solution	0	0	0	0	0	0	1.0 ± 0.0	1.0 ± 0.0	1.0 ± 0.0
Marketed LW/E	0	0	0	0	0	0	1.0 ± 0.0	1.0 ± 0.0	1.0 ± 0.0
Dextrose 5% and Sodium Chloride 0.45% Injection (406 mOsm/L)	1.0 ± 0.71	0	0	0	0	0	1.2 ± 0.5	1.0 ± 0.0	1.0 ± 0.0
Dextrose 10% *w*/*v* Solution (505 mOsm/L)	1.6 ± 0.6	0	0	0	0	0	1.6 ± 0.55	1.0 ± 0.0	1.0 ± 0.0
Sample 3A	1.4 ± 0.6 *	0.6 ± 0.5	0	0.6 ± 0.5	0	0	1.0 ± 0.5 ***	1.0 ± 0.0	1.0 ± 0.0
Sample 3B	1.4 ± 0.9 *	0	0	0.8 ± 0.5	0	0	2.0 ± 0.7 ***	1.2 + 0.5	1.2 + 0.5
Sample 3C	1.6 ± 0.6 **	0	0	0.6 ± 0.6	0	0	1.4 ± 0.6	1.2 + 0.5	1.2 + 0.5

ns: no significant difference, * *p* < 0.05 moderate difference; ** *p* < 0.01 strong difference; *** *p* < 0.001 very strong difference.

## Data Availability

Data is contained within the article.
